# Angular Oscillation of Solid Scatterers in Response to Progressive Planar Acoustic Waves: Do Fish Otoliths Rock?

**DOI:** 10.1371/journal.pone.0042591

**Published:** 2012-08-09

**Authors:** Petr Krysl, Anthony D. Hawkins, Carl Schilt, Ted W. Cranford

**Affiliations:** 1 University of California San Diego, La Jolla, California, United States of America; 2 Loughine Ltd, Kincraig Blairs, Aberdeen, United Kingdom; 3 Bigleaf Science Services, North Bonneville, Washington, United States of America; 4 Quantitative Morphology Consulting, Inc., San Diego, California, United States of America; University of Zurich, Switzerland

## Abstract

Fish can sense a wide variety of sounds by means of the otolith organs of the inner ear. Among the incompletely understood components of this process are the patterns of movement of the otoliths vis-à-vis fish head or whole-body movement. How complex are the motions? How does the otolith organ respond to sounds from different directions and frequencies? In the present work we examine the responses of a dense rigid scatterer (representing the otolith) suspended in an acoustic fluid to low-frequency planar progressive acoustic waves. A simple mechanical model, which predicts both translational and angular oscillation, is formulated. The responses of simple shapes (sphere and hemisphere) are analyzed with an acoustic finite element model. The hemispherical scatterer is found to oscillate both in the direction of the propagation of the progressive waves and also in the plane of the wavefront as a result of angular motion. The models predict that this characteristic will be shared by other irregularly-shaped scatterers, including fish otoliths, which could provide the fish hearing mechanisms with an additional component of oscillation and therefore one more source of acoustical cues.

## Introduction

Fish can sense and respond to a wide variety of sounds. They use sounds to communicate with one another, detect prey and predators, navigate from one place to another, avoid hazards, and analyse the world around them (see Ref. [1] for a recent review). The means by which fish detect sounds involve the otolith organs of the inner ear and are relatively well understood. Sound detection mechanisms in fish have been comprehensively reviewed by Popper et al [2]. However, elucidating the way these organs analyse sound quality and in particular how they enable fish to discriminate between sounds from different directions has proved more difficult.

The motion of the otolith organs of the white seabass (*Atractoscion nobilis*), a species of croaker, was studied in [3] using a finite element model of the three pairs of otoliths embedded in a viscoelastic matrix (tissue) and exposed to pressure waves in water [4]. The model otoliths were observed to execute both linear translation and angular oscillation, which we have called “rocking”. Subsequently we decided to use similar modeling tools to examine whether the angular motion was also to be observed in rigid scatterers in water as opposed to in an elastic matrix. An experimental study was also initiated in parallel by an independent research team [5]. The present work describes the model of the motion of rigid scatterers in acoustic fluids in response to progressive harmonic waves. The motivation for this work derives from the possibility that the angular motion of the otoliths may be an additional acoustical cue that the fish may use to process sounds.

The traditional view for sound detection proposes that fish tissue has similar acoustic properties (in terms of density and elasticity) to the surrounding water. When the fish is moved back and forth by a sound field, the dense otoliths within the ear lag behind because of their inertia, thereby creating shearing forces at sensory hair cells [6], [7]. Each hair cell is directional in its response to mechanical stimulation [7], [8].

A simple mathematical model of the otolith and its suspension was put forward by de Vries [9], [10]. He suggested that the movement of the otolith is critically damped, with a low natural frequency of vibration. A critically damped oscillator has a nearly constant response to a broad range of frequencies. However, the amplitude of motion will decline steeply above the natural frequency, causing a reduction in sensitivity to higher frequency vibrations. Sand and Karlsen [11], [12] have pointed out that such a system is essentially an array of accelerometers. The otolith organs are inherently sensitive to the kinetic sound component, particle velocity or its time derivatives particle acceleration or particle displacement, and not sound pressure [13], [14].

There is an issue over the determination of the direction of a sound source by fish. The ability of fish to discriminate sounds from different directions is clear, but the mechanism by which they achieve this has yet to be elucidated (see review in Ref. [15]). It has been established that teleost fish are able to discriminate between spatially separated sources under far-field conditions, both in the horizontal [16]–[18] and vertical [19] planes. Indeed, they are able to distinguish between sound sources at different distances [20]. This ability not only enables fish to locate the sources of sound but may also assist them in discriminating sounds from a particular source against the general non-directional noise background.

How then do fish determine the direction of a sound source? One of the obstacles in the way of understanding the mechanisms for determining sound direction is our lack of knowledge of the patterns of movement of the otoliths themselves. Is the simple two-dimensional accelerometer model proposed by de Vries [9], [10] adequate to explain the motion of the otolith, or are the movements more complex? How does the otolith organ respond to sounds from different directions? Sand and Michelson [21] examined the motion of different parts of the saccular otolith of the European perch (*Perca fluviatilis*). Using a tray attached to a horizontal shaker to hold the preparation and a laser vibrometer to detect otolith motions. They observed that vibration of the fish in the horizontal plane along its long axis resulted in vertical movements of both ends of the otoliths at several driving frequencies. An area of minimum vertical movement appeared around the midpoint of the otolith at different frequencies, indicating the existence of a horizontal axis of rotation or rocking. The results suggested a frequency dependent pattern of otolith rotational movement as a result of linear horizontal translation.

In the present work we attempt to understand variability in the motion of the otolith by studying the response of dense rigid scatterers suspended in an acoustic fluid to acoustic planar progressive waves. This problem was studied for the sphere by Hickling and Wang [22]. Both steady-state forces and torques exerted on elastic objects were studied by Smith [23], but the resulting motion was not addressed. This aspect was further investigated by Olsson [24], with the limitation that time-harmonic excitation was considered for spheroidal objects, but even though the possibility of angular oscillation was accounted for in the model it was not studied. Also, multiple scattering and multiple scatterers were not accommodated in the model. Fan et al. [25] studied acoustic (steady-state) radiation torques on arbitrary scatterers, but the resulting motion was not analyzed.

In this work we present an approach that allows us to study the motion of rigid scatterers suspended in an acoustic fluid in response to acoustic incident long-wavelength planar progressive waves and (multiple) scattered waves. First, in Section 2, we formulate a qualitative mechanical model for the motion of such scatterers. The model predicts that the motion will include both translational and angular oscillation, provided the scatterer is sufficiently asymmetric. In Section 3, we detail an acoustic time-domain finite element model. Verification of the model is undertaken in Section 4 where we report simulations of the response of a spherical scatterer for which the reference solution exists. The model is validated in Section 5 using recent experiments of Rodgers [5]. The hemispherical scatterer is found to oscillate both in the direction of the progressive waves and also in the plane of the waves as a result of angular oscillatory motion. Finally, in Section 6 we describe some numerical experiments that illustrate the rocking motion phenomena. Importantly, our model predicts that a rocking motion will be displayed by all irregularly shaped scatterers including fish otoliths. It is therefore possible that hearing mechanisms in fish could make use of this additional component of oscillation.

## Methods

### Qualitative Model of Motion

#### 1.1 Setting and assumptions

Consider a progressive planar harmonic wave in an acoustic fluid (in this paper we are mostly concerned with water) with mass density 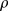, speed of sound 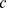, and angular frequency 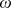. The acoustic wave impinges upon a stiff (so stiff that it may be considered rigid) homogeneous scatterer of arbitrary shape, whose characteristic dimensions 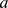 are all much smaller than the wavelength of the incident acoustic wave (it is “acoustically small”)


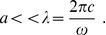
(1)

Expressed in the terms of the wavenumber 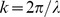, this assumption reads 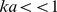. In particular, for an otolith in water we have 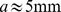 and 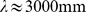 (for a 500 Hz signal), and the assumption is quite adequate.

**Figure 1:**
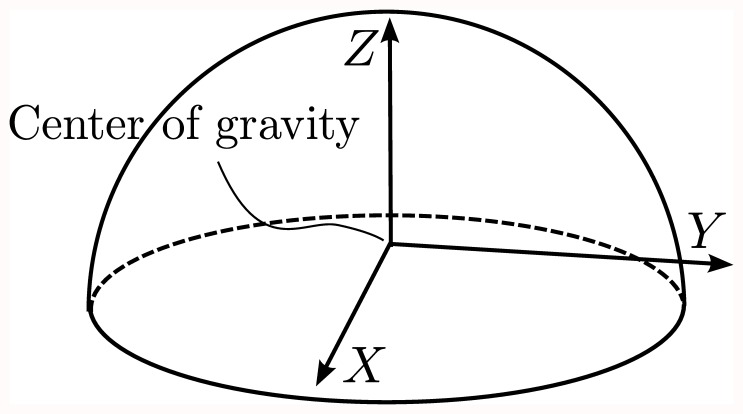
The geometry of the hemispherical scatterer.

Without any loss of generality, we will assume that the pressure is described in Cartesian coordinates such that the incident plane wave front is orthogonal to the *X* axis, and the center of the Cartesian coordinates is at the center of gravity of the scatterer. As a consequence of the assumption (11) the incident pressure, the scattered pressure, and their sum, the total pressure, around the scatterer at any time may be approximated by a Taylor series in coordinates *X,Y,Z*. To a first approximation we will limit the Taylor series to include only the constant and linear terms. For the pressure around the scatterer we write


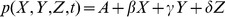
(2)

where all the coefficients of the Taylor series are functions of time, 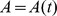, and so on. We will symbolically refer to the coefficients of the Taylor series as 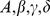 and distinguish their meaning by explicitly specifying whether the expansion is for the incident pressure or the total pressure.

#### 1.2 Resultant force

Henceforth we omit the time argument, with the understanding that the force changes in time. The force exerted upon the scatterer by the pressure in the surrounding fluid can be evaluated at any time as


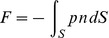
(3)

where *S* is the surface of the scatterer, and *n* is the outer normal to the surface. We will write 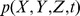 and 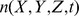 only when confusion is possible; otherwise the arguments will be omitted. The time-dependent force *F* causes the scatterer to accelerate in response to the forcing by the pressure according to Newton’s equation.

Substituting (2) into (3) we obtain the force *F* in the form of


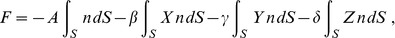
(4)

where we may now introduce a particular shape of the scatterer surface to evaluate the individual integrals.

### Resultant Torque

The torque exerted upon the scatterer by the pressure in the fluid surrounding it can be evaluated at any time as


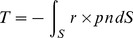
(5)

where 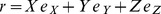 is the location where the pressure acts on the surface of the scatterer, and 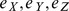 are unit basis vectors of the Cartesian coordinates. Equation (5) may be rewritten using the expansion of the cross product as


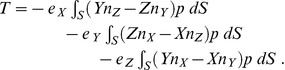
(6)

Upon substitution of (2) the individual integrals read


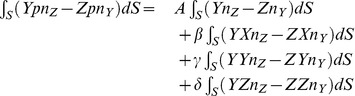
(7)

and


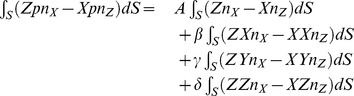
(8)

and finally
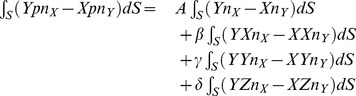
(9)


### Total Response Decomposition

The total pressure is composed of the incident pressure and the scattered pressure. Consequently, we may evaluate the total response of the scatterers as a superposition of the response due to the incident pressure and the response due to the scattered pressure. We will now apply this principle to the calculation of the dynamic force and torque acting on the solid scatterer.

**Figure 2:**
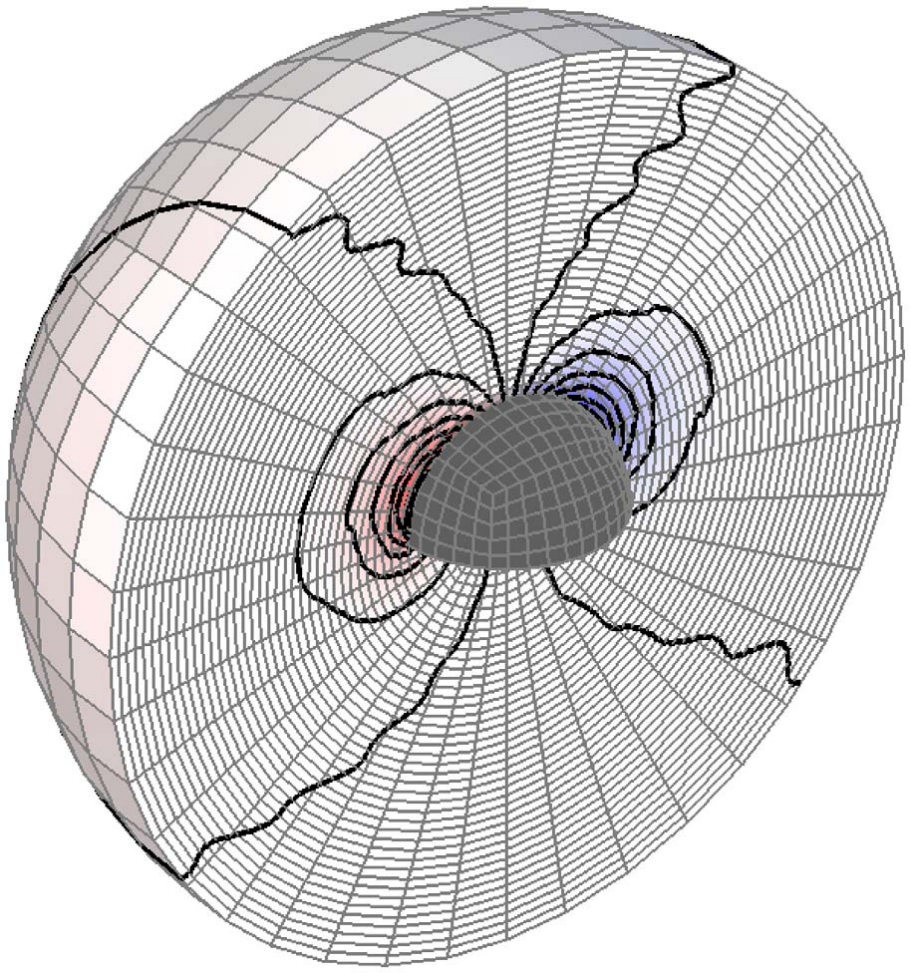
Perturbation (scattered) pressure distribution around the hemispherical scatterer at a particular time instant. The scatterer is the void in the middle. Planar incident wave of 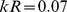. The pressure amplitude is coded by shade, the darker the shade the higher the positive (red) or negative (blue) pressure. Level curves of pressure are also shown.

**Figure 3:**
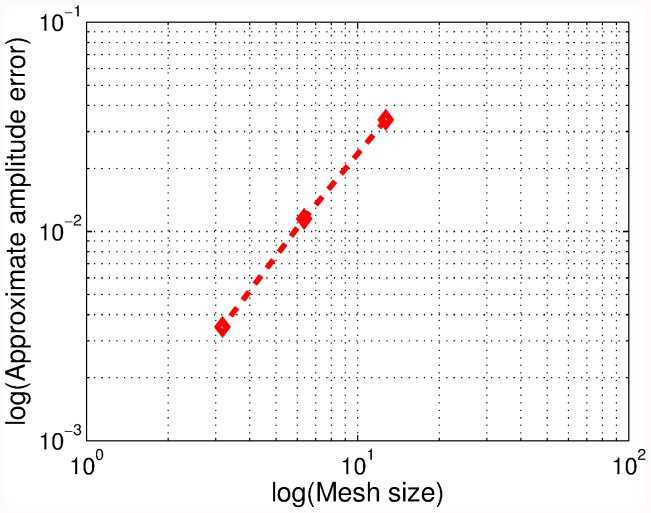
Approximate error of the normalized displacement amplitude for the spherical scatterer.

First we consider the effect of the uniform incident pressure. It is readily demonstrated that the first integral on the right of (4) due to uniform pressure does not contribute to the force no matter what the shape of the surface of the scatterer. We can write


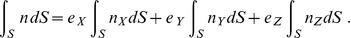
(10)

Consider a surface element 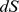. According to the well-known tetrahedron argument [26], we have the relationship between the projection of the elementary surface area into the plane 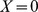



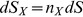
(11)

and similarly for the other two coordinate planes 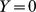 and 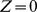



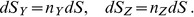
(12)

Now, because the surface *S* is closed, each elementary surface 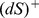 may be paired with another elementary surface 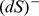 such that 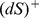 and 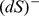 project into the same-size area in the plane 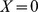



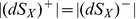
(13)

and the components of the normals to the corresponding elementary surfaces have opposite signs


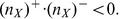
(14)

Analogous arguments can be made for projections into the coordinate planes 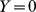 and 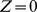. Since these observations are true for all elementary surfaces 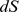, the contributions to the integrals from all such pairs will cancel and we have


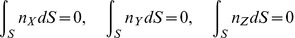
(15)

and consequently


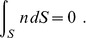
(16)

This finding demonstrates the conclusion that uniform pressure in the acoustic fluid would not accelerate the embedded scatterer.

### Spherical Scatterer, Response to Incident Pressure

Next we will evaluate the force due to incident pressure for a spherical scatterer of radius *R*. The coordinate planes 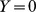 and 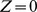 are planes of symmetry for the pressure on the surface *S*. Consequently the Taylor series expansion of the pressure will be represented by zero coefficients, 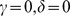, and the force will be evaluated as


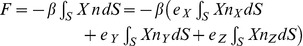
(17)

Similar arguments as above for the uniform pressure term can be applied. For instance,


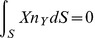
(18)

because for any pair of elementary surfaces such that 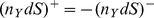 the corresponding coordinate 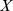 is the same for both, and we again get cancellation among all the pairs. Equation (17) for the force that acts on a spherical scatterer therefore reduces to


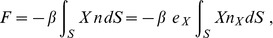
(19)

where the integral is evaluated over the surface of the sphere. The result


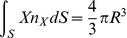
(20)

reproduces the observation that for acoustically small spheres (see equation (1)) the accelerating force will be proportional to the product of the average pressure gradient (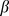) and the volume of the sphere.

The dynamic torque for a spherical scatterer is always zero, under all conditions, not only for the incident pressure, but also for the total pressure, as all the elementary pressure forces pass through the center of the sphere (the center of gravity).

**Figure 4:**
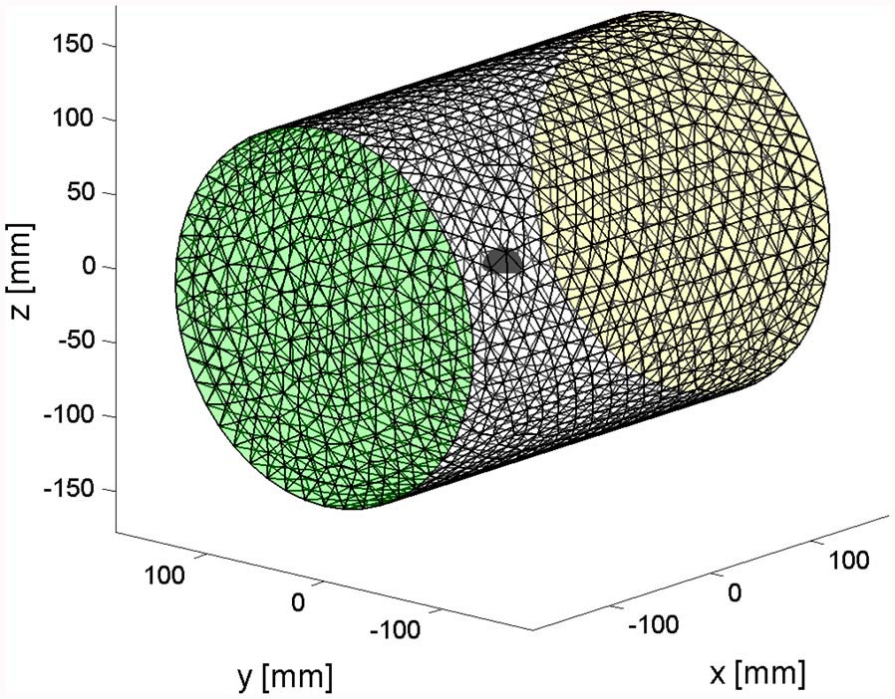
Mesh of the hemispherical scatterer located in the tank. Mesh size of 2 mm on the surface of the scatterer.

**Figure 5:**
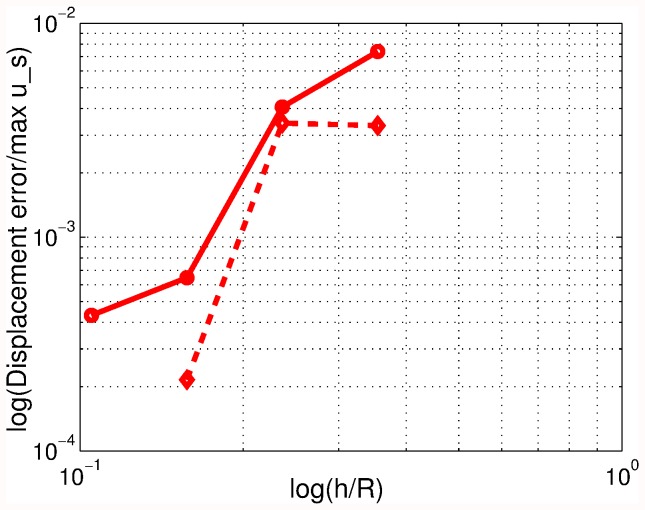
Approximate error (dashed line) and estimated true error of the longitudinal displacement of the hemispherical scatterer for the finite element solutions for excitation frequency of 100 Hz.

### Hemispherical Scatterer, Response to Incident Pressure

Now consider a hemispherical scatterer of radius *R* shown in Figure 1. Note that the center of gravity of the hemisphere is at a distance of 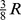 from the center of the spherical surface. Only the coordinate plane 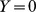 is a plane of symmetry for the pressure on the scatterer surface *S*. The Taylor series expansion of the incident pressure will contain no contribution of the linear term in the *Y* direction, 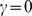, and no contribution of the linear term in the *Z* direction, 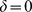. The force will be therefore evaluated as


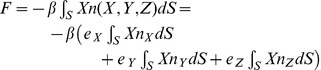
(21)

A similar argument as above for the uniform pressure term can be applied. Hence, we conclude that the accelerating force on the hemispherical scatterer will be in the form


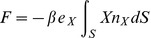
(22)

Therefore the scatterer will be accelerated by the incident pressure into oscillation only in the direction of the wave propagation. The integral evaluates to


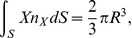
(23)

and again this finding is in agreement with the observation that the accelerating force will be proportional to the product of the average pressure gradient and the volume of the scatterer.

We shall now evaluate the dynamic torque due to the incident pressure on a hemispherical scatterer with the Cartesian coordinates origin located at the center of gravity (Figure 1). All of the integrals that define the torque components for the linearly varying incident pressure (2) in the immediate vicinity of the scatterer can be shown to be identically zero. This follows from the Gauss-Ostrogradski theorem


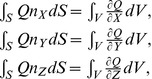
(24)

where upon substitution of a quadratic expression in the coordinates with the origin at the center of gravity, for instance 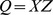, we find


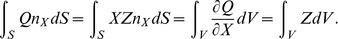
(25)

The rightmost integral is the definition of the static moment with respect to the plane 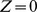. Recall that 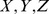 are Cartesian axes at the center of gravity, and all such static moments are by definition zero. For the same expression we also obtain


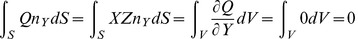
(26)

and


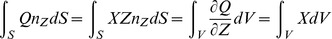
(27)

which is identically zero by the same static-moment argument. All other combinations are investigated analogously. Therefore, this argument shows that to first-order the incident pressure does not produce any dynamic torque on a scatterer of arbitrary shape.

### Hemispherical Scatterer, Response to Scattered Pressure

The scattered pressure distribution is not known analytically. As shown by theoretical argument (for instance in [27]) for low frequencies (small 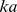) the scattered (perturbation) pressure will be similar to that generated by a translating object in fluid, and hence dipole-like. See Figure 2 for an illustration of the perturbation pressure as computed numerically for a hemispherical scatterer. Note this is just a snapshot from a time-dependent process. For any direction in the plane *X,Y* this picture would be similar as the scatterer is axially symmetric with respect to the axis *Z*. The pressure distribution would evidently change if the symmetry was broken such as for the sound fronts being oriented arbitrarily with respect to *X,Y,Z*.

**Table 1:** Comparison of simulations with experiments [5].

Quantity	Frequency [Hz]	Experiment	Model
Longitudinal displacement [ 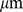 ]	100	3.33	2.98
	200	0.61	0.57
Rocking/longitudinal displacement ratio	100	7.5%	12.8%
	200	9.8%	12.8%

Hemisphere.

**Table 2:** Comparison of simulations with experiments [5].

Quantity	Frequency [Hz]	Experiment	Model
Longitudinal displacement [ 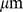 ]	100	3.38	3.08
	200	0.62	0.59
Rocking/longitudinal displacement ratio	100	9.5%	8.8%
	200	9.4%	8.8%

Hemicylinder of 4 inch length.

In the first approximation we may take a distribution of the scattered pressure that varies along the surface *S* as the projection of the translation due to the incident pressure along the *X* axis onto the outer normal to the surface *S*

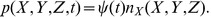
(28)


The torque components due to this scattered pressure distribution are


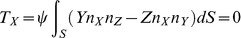
(29)

and


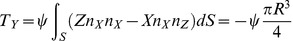
(30)

and finally


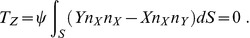
(31)

Clearly, the scattered pressure generates a nonzero dynamic torque about the *Y* axis. Therefore we conclude that our simple model predicts harmonic rocking (wobbling) of rigid scatterers in incident planar harmonic sound waves. In the following sections we will illustrate this point with a numerical investigation using the finite element method.

**Figure 6:**
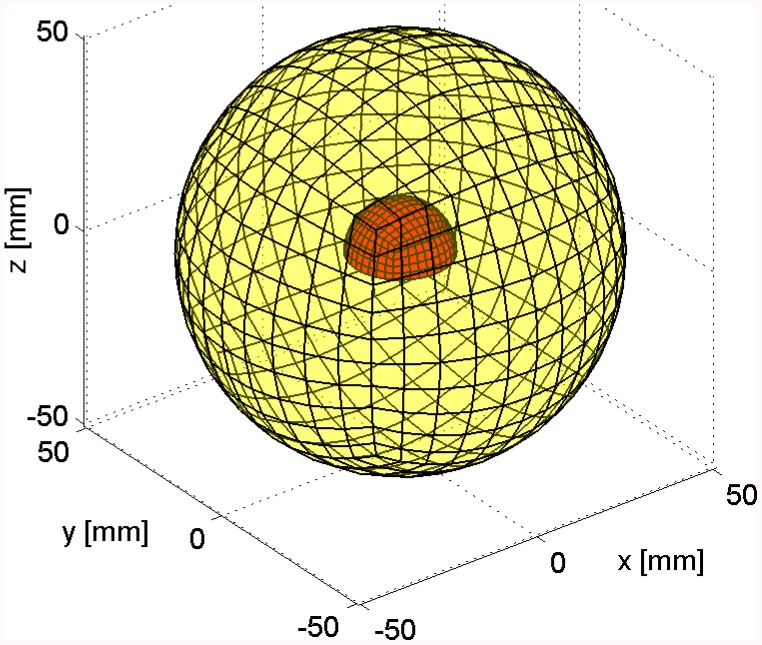
Simulation of hemispherical scatterer. Illustration of the mesh with 8 element edges per radius on the surface of the domain; the surface of the scatterer is shown in solid (red) color, and the outer surface with the absorbing boundary condition is transparent (yellow).

### Finite Element Model

The equation for the acoustic pressure


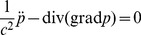
(32)

is treated with the Galerkin approach to yield the weighted residual equation


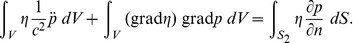
(33)

Here 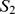 is part of the boundary of the volume *V* where the normal component of the pressure gradient is prescribed, and 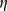 is the test function which vanishes on the part of the boundary 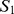 where the pressure is prescribed.

The total pressure *p* is decomposed into the pressure of the known incident wave 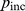 and the unknown perturbation (scattered) pressure *P* as 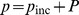, and the appropriate boundary conditions for the constituent parts are formulated next. The problem is solved in a volume of acoustic fluid *V* of finite extent. The computational volume *V* encloses the scatterer. First we consider a rigid simply-connected scatterer of mass density 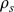, volume 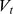, and surface 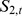. The surface of the computational domain is divided into the part where the fluid is adjacent to the enclosed rigid scatterer 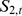 and the part where the fluid inside the computational domain is separated from the infinite extent of fluid exterior to the computational domain 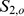
_._

**Table 3:** Hemisphere in infinite fluid medium.

Quantity	Frequency [Hz]	Model
Longitudinal displacement [ 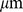 ]	100	3.184
	200	0.598
Rocking/longitudinal displacement ratio	100	13.4%
	200	13.4%

**Table 4:** Hemicylinder of 4 inch length in infinite fluid medium.

Quantity	Frequency [Hz]	Model
Longitudinal displacement [ 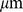 ]	100	3.389
	200	0.643
Rocking/longitudinal displacement ratio	100	14.8%
	200	14.8%

On the surface of the rigid body scatterer 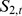 the *total* pressure *p* satisfies the boundary condition


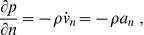
(34)

where 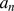 is the acceleration of the surface of the scatterer in the direction of the outer normal to the fluid surface *n* due to the motion of the scatterer. This may be expressed in terms of the acceleration of the center of gravity of the scatterer *A*, and the angular acceleration of the scatterer about its center of gravity 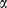



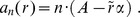
(35)

Here *r* is the position vector of a given point on the surface of the scatterer relative to its center of gravity, and 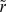 is the skew-symmetric matrix used to express a vector cross product


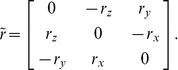
(36)

The weighted residual equation boundary term on the surface of the scatter therefore reads


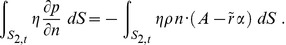
(37)

On the surface 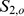 where the fluid extent is truncated the boundary conditions are applied separately to the incident pressure and the perturbation pressure. For the incident pressure the pressure gradient is known. For instance, for a planar harmonic wave we have


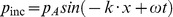
(38)

and therefore the gradient of the incident pressure on 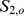 reads


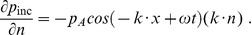
(39)

Absorbing (radiation) boundary condition is applied to the perturbation pressure. The simplest such boundary condition is the early-time plane wave approximation


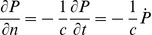
(40)

for the perturbation pressure on 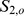, and that was found sufficient for the present application: the convergence rate is apparently affected but remains satisfactory.

The weighted residual statement is written with the above boundary conditions as


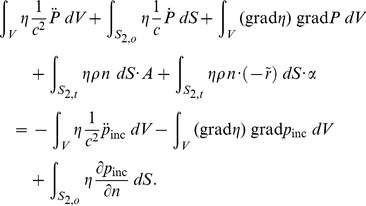
(41)

To eliminate the translational and angular acceleration of the scatterer we invoke Newton’s equation: acceleration *A* may be written in terms of the mass of the scatterer 
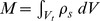
 and force applied on its surface


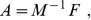
(42)

where the force applied on the scatterer *F* is produced by the total pressure on the surface of the scatterer


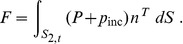
(43)

Here *n* is the *outer* normal to the fluid-scatterer boundary with respect to the solid. Similarly the angular acceleration of the scatter about its center of gravity may be written in terms of its tensor of inertia and the applied torque


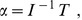
(44)

where the torque applied on the scatterer *F* is produced by the total pressure on the surface of the scatterer


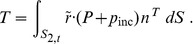
(45)

Here the tensor of inertia is


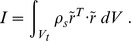
(46)

Substituting for the accelerations from (42) and (44) into (41) results in a weighted residual equation for the perturbation pressure as the only unknown. Classical finite element discretization (with iso-parametric hexahedra, or tetrahedra, in the present case) allows us to write the matrix equations for a system of ordinary differential equations for the nodal pressures


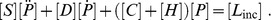
(47)

Here 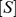 is the acoustic stiffness matrix, 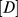 is the absorbing-boundary condition damping matrix, 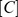 is the acoustic mass matrix, and 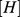 is defined as


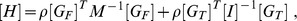
(48)

where the coupling matrices 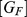 and 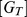 implement the discrete form of (42) and (44)


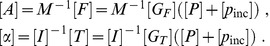
(49)

The load vector consists of.


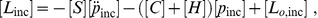
(50)

where 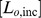 is the matrix expression of the incident-pressure load term on 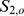. Using the definition


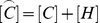
(51)

allows us to rewrite (47) as a system of coupled first order differential equations


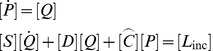
(52)

Straightforward discretization in time with the trapezoidal rule yields


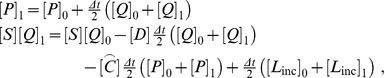
(53)

which results in the time-stepping algorithm: (a) solve for perturbation pressure rate


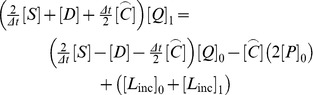
(54)

then (b) update perturbation pressure


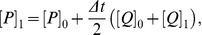
(55)

and finally (c) evaluate the accelerations from (49) and integrate to obtain velocities and displacements. To address the possibility of the presence of multiple scatterers we may realize that the above argument applies separately to each of the scatterers, with the understanding that the perturbation pressure 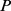 accounts for the presence of all scatterers. Therefore the computation is straightforward and the only additional effort involves the construction of the multiple coupling matrices 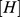 and the update of the accelerations for each scatterer. This computational scheme was implemented in the Matlab toolkit FAESOR [26].

## Results

### 1. Verification

The response of a movable spherical scatterer was studied in detail by Hickling and Wang [22]. The amplitude of the displacement in the direction of the progressive wave is predicted by their equation (5), with the displacement function given for the large wavelength limit in equation (3) of the above reference. Here we predict the motion of the spherical scatterer with the finite element model described above. The computational domain was a sphere of water 15 mm in radius (bulk modulus 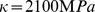, mass density 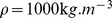). The spherical scatterer with radius of 5 mm of mass density 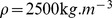 was embedded in the center of the domain. The finite element mesh was structured, and composed of eight-node hexahedra.

**Figure 7:**
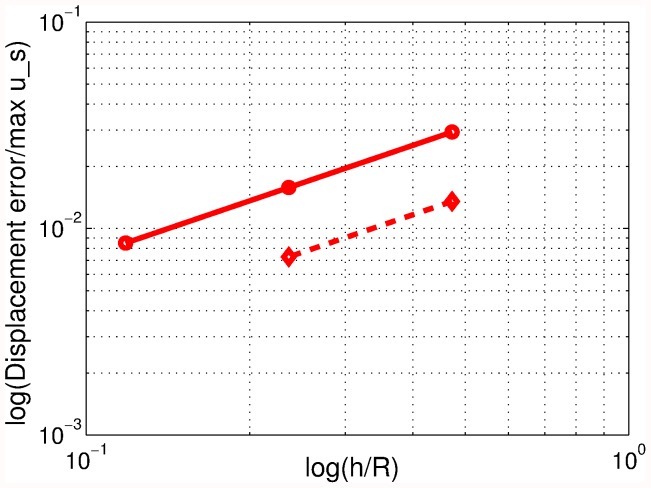
Approximate error (dashed line) and estimated true error of the longitudinal displacement of the hemispherical scatterer for the finite element solutions for excitation frequency of 100 Hz.

**Figure 8:**
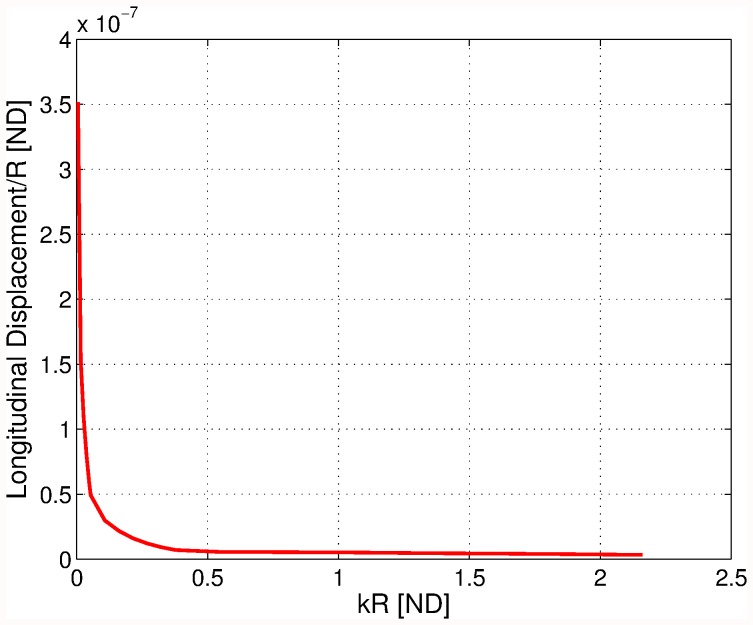
Longitudinal displacement normalized by the radius of a hemisphere scatterer as a function of frequency (in terms of the parameter 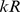).

The scatterer was initially at rest, and the progressive planar harmonic waves in the positive *X* direction, with incident pressure amplitude of 1 kPa, were introduced with frequency of 100 Hz. The model was also exercised with other (long-wavelength) frequencies, without significant difference in the match between the analytical and numerical predictions. The computed dynamic response was post-processed to extract the amplitude of the steady-state motion of the sphere.

**Figure 9:**
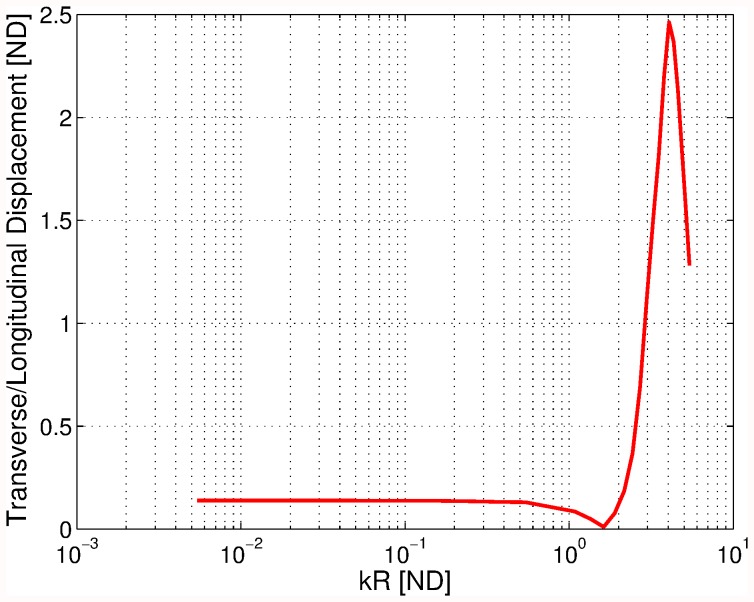
Ratio of transverse to longitudinal displacement of a hemisphere scatterer as a function of frequency (in terms of the parameter 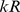).

The *X* displacement (in the direction of the progressive wave) of the center of gravity is normalized by the displacement 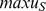 computed for a spherical scatterer in [22]


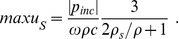
(56)

The computed results are used to define the approximate error of the displacement amplitude as


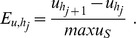
(57)

Figure 3 demonstrates the convergence of the finite element approximation. The normalized displacement amplitude was computed for four meshes, with two, four, eight, 16 element edges per radius as 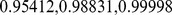 and 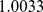. The log-log plot of the approximate errors shows satisfactory convergence rate of approximately 1.7, and the finest mesh renders the motion amplitude within 1% error.

### Validation

An experimental study to detect and quantify the angular motion of rigid scatterers in water was recently reported [5]. Aluminum scatterers were suspended submerged in a cylindrical tank with piston-generated standing waves (this rig approximated the conditions of an unsuspended scatterer in infinite medium). The longitudinal and angular oscillatory motion was measured with a variety of techniques. While the experimental study [5] was ongoing we were supplying the experimenters with blind predictions. Here we reproduce the results for the hemispherical scatterer and for one hemicylinder configuration.

In the simulations, the sound speed of water was taken as 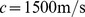, and mass density as 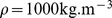. The hemispherical aluminum scatterer with radius of 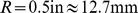 and the hemicylinder aluminum scatterer with radius of 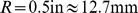 and length 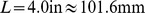, of mass density 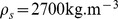, were suspended at the depth of 7″ (i. e. 177.8 mm, halfway between the piston and the free surface) in the cylindrical tank.

The excitation signals of 200 Hz and a pressure amplitude of 738.2 Pa, which corresponds to the experimental measurement of 261 Pa RMS at 7 inch depth, and of 100 Hz and a pressure amplitude of 973.0 Pa, which corresponds to the experimental measurement of 344 Pa RMS at 7 inch depth, were considered.

#### 1.1 Hemispherical scatterer

Unstructured graded (four-node) tetrahedral meshes with mesh size on the surface of the scatterer of 1.33, 2.0, 3.0, and 4.5 mm were used. One such mesh is shown in Figure 4 where the surface of the hemispherical scatterer is enclosed within the transparent surface of the volume of the water.

The four finite element solutions for the displacement and angular rotation were used in a Richardson’s extrapolation procedure [26] to extract the limit values. For the longitudinal displacement (in the direction of the propagation of the wave) two successive solutions yielded the normalized approximate error as


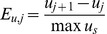
(58)

where 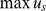 is the displacement amplitude of an equivalent spherical scatterer from [22], and 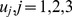 are the numerical solutions. The true error was then estimated from the relation (12.17) of [26]


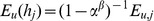
(59)

where 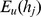 was the true error for mesh size 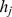, 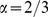 was the refinement factor, and 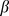 was the rate of convergence, which had to be estimated theoretically as the data was not sufficiently smooth for a reliable least-squares estimate of the convergence rate (as shown in Figure 5). In this instance, we assumed 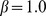 due to the use of the first order absorbing boundary condition.

The computed amplitude of total displacement in the direction of the wave propagation (longitudinal) and rocking displacement (amplitude of displacement perpendicularly to the flat base of the scatterer) is reported in Table 1. The predicted longitudinal displacement for both excitation frequencies can be seen to be quite close to the experimental measurements. The agreement of the ratio of the rocking/longitudinal displacement is, for reasons incompletely understood at this point, not as good. Perhaps the suspension of the hemispherical scatterer somehow prevented the transverse displacement to some degree.

### Hemi-cylindrical Scatterer

Three lengths 4′′, 6′′, and 8′′ respectively, of hemi-cylindrical rods were tested [5]. For the sake of brevity we report comparison for just the 4′′ configuration. The same material properties and pressure gradients as for the hemispherical scatterer were assumed. Similar approach to the limit procedure as for the hemispherical scatterer was adopted to produce results reported in Table 2. The agreement of the longitudinal displacement and of the ratio of the rocking/longitudinal displacement for both excitation frequencies can be seen to be quite good.

### Hemispherical Scatterer in Infinite Medium

The experimental rig aims to produce conditions similar to a free-floating scatterer in infinite acoustic medium. In the previous sections we reported simulations that emulate the actual boundary conditions of the cylindrical tank with a piston at the bottom and a free surface of the top. Here we report simulations for scatterers placed in an infinite extent of acoustic fluid. First for the hemispherical scatterer, and then for the hemicylindrical scatterer.

The computational domain was a sphere of water of the radius 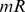 with absorbing boundary condition, where *m* needed to be chosen judiciously to reduce the effect of the boundary of the domain on the solution. It was determined by experimentation that taking the volume of water to be a sphere of radius four times the radius of the scatterer (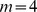) reduced the boundary effect to the fourth significant digit.

Hexahedral meshes with 9, 14, and 20 edges per radius were used. The meshes were structured and therefore the mesh size increased with distance from the surface of the scatterer: Compare with a sample mesh in Figure 6. The hemispherical scatterer’s surface is shown in solid color, and the outer spherical surface on which the absorbing boundary conditions are applied is shown transparent.

The excitation signal of the incident planar wave was considered at 200 Hz with a pressure amplitude of 2.478 kPa (corresponding to experimental measurement of 261 Pa RMS at 7 inch depth), and at 100 Hz and a pressure amplitude of 6.532 kPa (corresponding to experimental measurement of 344 Pa RMS at 7 inch depth).

The computed total displacement in the direction of the wave propagation (longitudinal) and rocking displacement (perpendicularly to the flat base of the scatterer) is reported in Table 3. The rocking to longitudinal displacement ratio is rather larger than for the tank configuration.

### Hemicylinder Scatterer in Infinite Medium

For the sake of brevity we report comparison for just the 4′′ long cylinder configuration. The same material properties and pressure gradients as for the hemispherical scatterer were used. The domain was an elongated ellipsoid that enclosed the scatterer with water of approximately the thickness of the diameter of the cylinder. The finite element model used an unstructured tetrahedral mesh (four-node elements). The extrapolated values of displacement computed from results for mesh size of 6.7, 4.5, and 3 mm are reported in Table 4 and the approximate and estimated true error of the longitudinal displacement are displayed in Figure 7. The predicted rocking motions are larger than those measured in the tank. This discrepancy is likely due to the difference of perturbation pressure distribution (which drives the rocking as opposed to the longitudinal motion driven by the incident field) between the free-space and the distribution in the experimental chamber of the finite-size cylinder.

### Rocking Experiments

Importantly, the present numerical formulation is not limited to large wavelengths, and that gives us an opportunity to investigate rocking as a function of frequency. For this investigation the computational domain was a sphere of water 15 mm in radius (bulk modulus 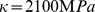, mass density 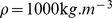). The hemispherical scatterer with radius of 5 mm and mass density 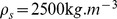 was embedded approximately in the center of the domain. The finite element mesh was structured, and composed of eight-node hexahedra.

The scatterer was initially at rest, and quickly (over a couple of periods) yielded to the excitation by the progressive planar harmonic waves in the positive *X* direction, with incident pressure amplitude of 1 kPa. The incident wave was applied with various frequencies (between 100 Hz and 100 kHz). The longitudinal displacement and the ratio of the transverse and longitudinal displacement were computed as in the previous section.

Figure 8 shows the change of the longitudinal displacement as a function of the wave number (frequency). The displacement can be seen to vary essentially smoothly as an inverse function of the wave number.

Figure 9 shows how the ratio of the transverse rocking displacement to the longitudinal displacement changes as the frequency was increased from low (very-long wavelength) to high (wavelength commensurate with the radius of the hemisphere). For low frequencies (large wavelengths) the transverse/longitudinal ratio was essentially independent of the frequency of excitation. A plausible explanation is that in this frequency range the perturbation pressure distribution does not substantially change, and its amplitude is proportional to the amplitude of the incident wave. For frequencies commensurate with the radius of the scatterer the rocking motion amplitude first seems to pass through a null at around 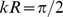 (i.e. 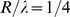), to subsequently increase to approximately a double of the longitudinal amplitude.

## Discussion

The present results indicate that the motion of complicated shapes such as otoliths under plane harmonic wave excitation is more complex than a simple back-and-forth oscillation in the direction of the progressive waves suggested by Pumphrey [6] and de Vries [9], [10]. A simple back and forth translation in the direction of the sound wave would only be the case for a uniform spherical otolith. If the shape of the otolith is irregular and asymmetrical, as it usually is, then motion is also induced in other directions. The rocking (or wobbling) motion may produce additional stimuli that the fish may be able to process for additional cues on the characteristics and direction of the oncoming sound.

For a simple hemisphere the rocking or wobbling motion is significant in magnitude (exceeding 10% of the back and forth oscillation). We might expect it to be much greater for some other shapes. In addition it is conceivable that the suspension system of the otolith in live intact fish may serve to accentuate the rocking motion. Certainly these possibilitites are worthy of further study.

There are more insights to be gained from the present simple models. For instance, how does the excitation direction relative to the orientation of the scatterer or otolith change the response? How does the response depend on the shape of the otolith? How do responses change for a grouping of otoliths (two or more), and how do the resulting motions depend on the excitation direction relative to the arrangement of the scatterers? There are many intriguing questions that may be amenable to closer examination with our models. In particular, the present finite element model can provide answers for multiple scatterers in arbitrary arrangements and allows for the actual otolith shapes to be modeled.

We must also not forget that the movements of otoliths are constrained within the otic capsule. Each otolith is surrounded by a fluid, the endolymph, and contained within a capsule. The otolith is attached to the wall of the capsule and placed in close contact with the hair cells of the sensory maculae by an otolithic membrane. These surrounding structures may also restrict and otherwise affect the motion of the otoliths in a way which we hope to investigate further.
